# Supplementary material for: How do people feel about sleep diaries? Factors influencing sleep diaries completion with and without daily exposure to light
Source: PLoS One. 2025 Mar 19;20(3):e0317788. doi: 10.1371/journal.pone.0317788 (PMC11922216; doi:10.1371/journal.pone.0317788)
Supplement: Attachment — Submitted filename: response to reviewers.docx [file pone.0317788.s001.docx]

**Response to reviewers**

*Dear reviewers,*

*We would like to thank you for your insightful feedback on our study. We are pleased to hear that you find our work on the use and improvement of sleep diaries valuable, particularly regarding the practical application of incorporating light exposure information. We appreciate your suggestions for enhancing our manuscript and have addressed your comments as follows.*

**Comment : there is room for improvement in the background introduction, methodological details, and discussion of results.**

1. **Background Introduction:**

*We have expanded the background section to provide a more comprehensive overview of the existing literature. We have also included a more detailed explanation of the rationale behind incorporating light exposure information into sleep diaries, supported by recent studies and relevant theoretical frameworks*.

2. **Methodological Details:**

*We have revised the methodology section to include more precise details about our study design, data collection procedures, and analysis methods. This includes a clearer description of the tools and techniques used for sleep diary. These additions aim to enhance the reproducibility and transparency of our research.*

3. **Discussion of Results:**

*We have enriched the discussion section by providing a more in-depth interpretation of our findings, highlighting the implications for both clinical practice and future research. Additionally, we have addressed potential limitations of our study and opened on suggestions for further investigation to build on our findings.*

**Comment: The study does not clearly explain how the reliability of the sleep diaries was validated.**

*We have added a detailed explanation in the introduction, methodology and discussion section regarding the reliability of the sleep diaries, as well as the steps od development of sleep light diary. We have also included references to studies that support the validity of the methods used.*

**Comment: The content and results of the study are somewhat unconvincing, especially given the low return rate of the diaries.**

*We acknowledge the concern regarding the low return rate of the diaries. The low return rate of the diaries is comparable to the low rates of adherence and compliance by patients to the care following a medical consultation. A number of measures could be implemented to improve this low rate: regular reminders, clear explanations, and verification of understanding, among others. We completed the discussion on this subject. We hope to be more convincing about the results.*

**Comment: It is recommended to increase the discussion on the limitations of the study, such as insufficient sample size and short follow-up duration, and propose directions for future research improvements.**

*We have expanded the limitations section to explicitly address the concerns about sample size and follow-up duration. We have proposed several directions for future research, including increasing sample size, extending the follow-up period, and exploring additional variables that may influence the outcomes.*

**Comment: Ensure the uniformity and accuracy of citation formatting.**

*We have thoroughly checked and revised the citation formatting throughout the manuscript to ensure uniformity and accuracy.*

*We believe these revisions have strengthened our manuscript and addressed the concerns raised. Thank you once again for your valuable feedback and for the opportunity to improve our work. We look forward to your further comments and hope that our revised manuscript meets your expectations.*

*Sincerely,*
