# Supplementary material for: How do people feel about sleep diaries? Factors influencing sleep diaries completion with and without daily exposure to light
Source: PLoS One. 2025 Mar 19;20(3):e0317788. doi: 10.1371/journal.pone.0317788 (PMC11922216; doi:10.1371/journal.pone.0317788)
Supplement: Attachment — Submitted filename: 2024_11_25_Responses_reviewers.docx [file pone.0317788.s002.docx]

**Response to reviewers**

*Dear Editor,*

*We would like to thank you for your interest in our work. We also express our gratitude to the reviewers of this second round, for their valuable comments and suggestions. They have helped improve this manuscript.*

*Please find below our responses to their comments and questions.*

*Sincerely,*

Reviewer #1 :

The authors acknowledge some limitations, but this section should also highlight the retrospective nature of the study and how it may have influenced the findings.

*Thank you for your comment. We apologize for the misunderstanding. This was indeed a prospective study. An empty sleep diary was sent to each patient (randomly assigned to either USD or LSD), and they were asked to fill it out daily until their next consultation. Sleep diaries were collected at that time and continued to be collected until the end of the study. The study is indeed prospective, with the sleep diary variable being randomized. Data regarding diary completion were gathered progressively during follow-up and do not represent pre-existing data. We have added a sentence to the Methods section to clarify this point (p7, line 116-119).*

The small number of correctly filled-out diaries and potential biases (e.g., patients more engaged with their treatment may have been more likely to return their diaries) should be elaborated on.

*We believe this point is addressed in the Discussion section. We focused on insomniac patients, who indeed completed the diaries more consistently than OSAS patients, for instance (see page 11). We have further elaborated on this point in the Discussion, specifically on line 304.*

Reviewer #2:

*Did the insomniac group returned sleep diaries in both USD and LSD group or just LSD-insomniac group? I think this is not clear just by reading the abstract (lines 37 and 40 in the R1 version w/ track changes).

*Both USD and LSD were returned. We have modified the abstract for greater precision.*

* To encourage light data in the sleep diaries, there is no need to underestimate the measurement of actimeters, since it is quantitative data which is actually more reliable than diaries (line 57 in the R1 version w/ track changes).

*Our intention was not to create this impression but rather to highlight the differences in accessibility between actimeters coupled with luxmeters. We have revised the Introduction section to make this clearer (p4, line 57).*

* Figure 1 a and b should have legends to explain them. Also, non-english terms should be replaced with english words. It is not clear what up and down arrows or filled circle etc mean for a person just looking at the figure.

*Thank you for pointing this out. We have updated the sleep diary to include an English version.*

* If figures are labeled as Fig.1a/b, they should be mentioned just like that at lines 99 and 104.
* Table 1, Table 3 and abbreviations say ‘UD’ which should be replaced with ‘USD’ to keep the consistency throughout the manuscript
* Line 289, ‘Study’ should be ’study’

*These items have been reviewed and corrected.*
